# Climate change, society, and pandemic disease in Roman Italy between 200 BCE and 600 CE

**DOI:** 10.1126/sciadv.adk1033

**Published:** 2024-01-26

**Authors:** Karin A. F. Zonneveld, Kyle Harper, Andreas Klügel, Liang Chen, Gert De Lange, Gerard J. M. Versteegh

**Affiliations:** ^1^ MARUM, Center for Marine Environmental Sciences, University of Bremen, Leobener Str. 8, 28359 Bremen, Germany.; ^2^ Geosciences Department, University of Bremen, Klagenfurter Str., 28359 Bremen, Germany.; ^3^ Department of Classics and Letters, University of Oklahoma, 650 Parrington Oval, CARN 110, Norman, OK 73019-4042, USA.; ^4^ Santa Fe Institute, 1399 Hyde Park Rd, Santa Fe, NM 87501, USA.; ^5^ Faculty of Geosciences, department of Earth Sciences, Geochemistry, University of Utrecht, Princetonplein 9, 3584 CC Utrecht, Netherlands.; ^6^ Department of Physics and Earth Sciences, Constructor University Bremen, Campus Ring 1, 28759 Bremen, Germany.

## Abstract

Records of past societies confronted with natural climate change can illuminate social responses to environmental stress and environment-disease connections, especially when locally constrained high–temporal resolution paleoclimate reconstructions are available. We present a temperature and precipitation reconstruction for ~200 BCE to ~600 CE, from a southern Italian marine sedimentary archive—the first high-resolution (~3 years) climate record from the heartland of the Roman Empire, stretching from the so-called Roman Climate Optimum to the Late Antique Little Ice Age. We document phases of instability and cooling from ~100 CE onward but more notably after ~130 CE. Pronounced cold phases between ~160 to 180 CE, ~245 to 275 CE, and after ~530 CE associate with pandemic disease, suggesting that climate stress interacted with social and biological variables. The importance of environment-disease dynamics in past civilizations underscores the need to incorporate health in risk assessments of climate change.

## Teaser

Southern Italian temperature and precipitation changes between 200 BCE and 600 CE can be linked with pandemic disease.

## INTRODUCTION

The ongoing accumulation of paleoclimate proxy records has enhanced the study of the resilience and susceptibility of human societies to climate change in past times (*1*, *2*)*.* It is widely considered that natural climate change can be associated with processes of social development and adaptation as well as crisis and collapse (*3*–*5*)*.* The climate system affected past human societies through a variety of direct and indirect mechanisms, from agricultural productivity (*6*) to perceptions of political legitimacy (*7*, *8*)*.* Of notable interest, not least due to the global impact of the COVID-19 pandemic, are potential links between climate change and dynamics of infectious disease (*9*, *10*)*.* These links are imperfectly understood, both because of the incompleteness of the empirical record and because of the complexity of the relationship between the physical climate and biological systems (*1*, *2*)*.* These general uncertainties extend to particular cases, such as the experience of the Roman Empire, whose time span overlaps some of the first widely attested pandemic disease outbreaks in human history, i.e., the Antonine Plague (~165 to 180 CE), the Plague of Cyprian (~251 to 266 CE), and the First Plague Pandemic (~541 to 766 CE, the first wave of which in the 540s is known as the Plague of Justinian).

While there is debate about the extent and mechanisms of the climate’s influence on social dynamics, there is agreement that regionally specific and high-resolution proxy records have the greatest potential to cast light on the impact of past climate change (*1*)*.* Despite a relatively well-developed literature on the impact of climate change in Roman times, analysis has been limited by the lack of reliable high-resolution paleoclimate records for Roman Italy (*4*, *11*–*13*)*.* Unfortunately, tree-ring series in Italy do not now reach back to the Roman period. Alpine dendrochronological records provide high temporal resolution but limited spatial correlation with parameters of interest in peninsular Italy (*3*, *14*)*.* Speleothem records generally have low chronological resolution and often lack the ability to disambiguate temperature and precipitation signals (*15*–*18*)*.* There are a number of detailed pollen series from ancient Italy, but these records have low temporal resolution or reflect strong anthropogenic influence (*19*–*22*). Hence, it has proven difficult to reconstruct the climate in the heartland of the Roman Empire.

**Fig. 1.**
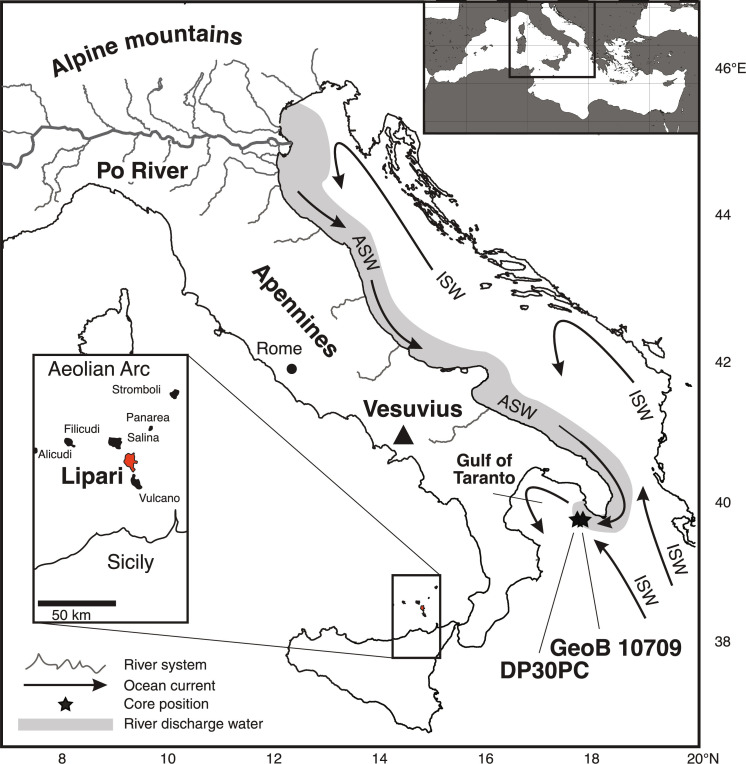
Map of Italy and the Adriatic Sea indicating the major river systems, marine surface water currents, core positions, and important geographic features. ISW, Ionian Surface Water; ASW, Adriatic Surface Water [redrawn after (*29*)]*.*

**Fig. 2.**
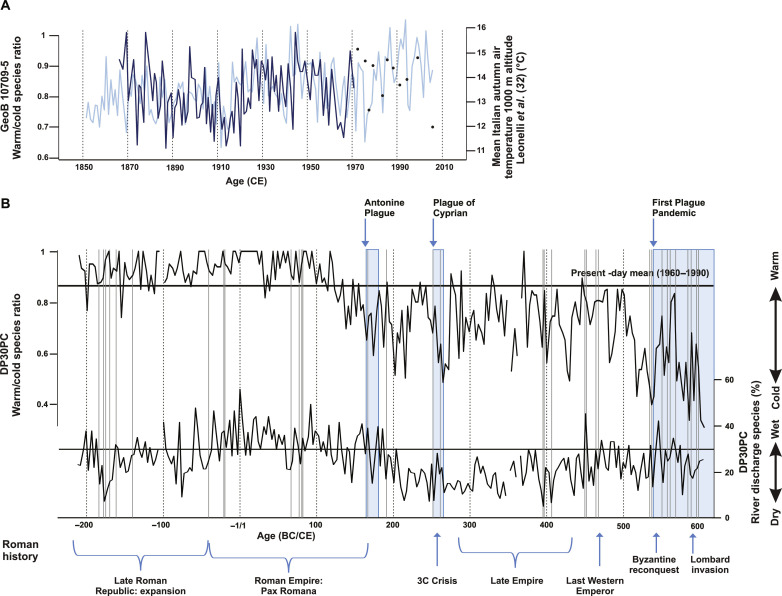
Reconstructed late-summer/autumn relative temperatures and precipitation. (**A**) Comparison between late-summer/autumn dinoflagellate cyst–based *W*/*C* ratio (black line + black points) of core GeoB 10709-5 and mean autumn Italian temperatures at 1000-m altitude (blue line). (**B**) Late-summer/autumn dinoflagellate cyst–based *W*/*C* ratio and relative abundance of discharge species (nutrient sensitive) reconstructions (black lines) and the occurrence of epidemics and pandemics in the Roman empire (blue blocks) as well as disease outbreaks in Roman Italy (gray lines) and major historical periods/events.

Here, we present a high–temporal resolution (one sample/~3 years of sediment deposition) reconstruction of Italian autumn temperature and precipitation from ~200 BCE to ~600 CE based on a marine archive from the Gulf of Taranto (Core DP30PC, 39°50.07 N, 17°48.05 E, water depth of 270 m; Fig. 1) and compare these with the occurrences of epidemics and pandemics in Italy (Fig. 2 and table S1). The chronology of DP30PC is based on ^210^Pb, ^14^C, and tephrochronology (see Materials and Methods). The site location of core DP30PC is positioned at the distal end of a river discharge plume that finds its origin in the north Adriatic Sea. The discharge waters [Adriatic Surface Waters (ASW)] originate from the Po River, smaller northern Italian rivers draining the southern face of the Alps, and rivers draining the north and eastern parts of the Apennines (Fig. 1) (*23*, *24*)*.* These nutrient-rich, low-saline waters mix with high-saline, oligotrophic waters (Ionian Sea Water) that have entered the Adriatic Sea in the southwestern side of the Gulf of Taranto. Surface water temperatures at the core location reflect southern Italian air temperatures (*25*), whereas upper water salinity and nutrient concentrations reflect the presence and amount of ASW that, today, has its maximal extensions in early spring and autumn due to the melting of snow and ice in the Alps and Apennines (spring) and enhanced precipitation (autumn). Local temperatures at the core site and precipitation in the Alps and Apennines are influenced by regional and extra-regional climate systems of which the North Atlantic Oscillation (NAO) is the most important (*26*)*.* Its positive and negative modes reflect the strengths of the high-pressure cell above the Azores and low-pressure cell above Iceland. The negative mode of the NAO is characterized by enhanced precipitation and relatively low temperatures in Italy and southwestern Europe (*27*)*.* Although temperatures and precipitation in Italy are locally constrained, the NAO has an extra-regional effect on the changes in these parameters, indicating that changes in southern Italian temperature reflect extra-regional temperature change. Climate systems such as the Western Mediterranean Oscillation and cold/dry southward blowing winds of the Bora as well as northward blowing warm/dry winds from the Sahara (Sirocco) influence regional Italian climate as well.

We established temperature and precipitation reconstructions by studying the downcore fossil dinoflagellate cyst association. Cyst-forming dinoflagellates are unicellular algae that live in the upper water column producing morphologic species-specific fossilizable cysts as part of their life cycle (*28*)*.* Their association composition reacts very sensitively to upper water environmental change such as upper water temperature and nutrient composition (*29*)*.* In the Gulf of Taranto, cyst production occurs mainly in late summer/autumn, causing the downcore cyst association composition to reflect changes in late summer/autumn conditions (*30*, *31*)*.* During this season, upper water temperatures correspond to air temperatures, whereas the salinity and nutrient concentration is determined by ASW that is steered by precipitation in the southern Alps and Apennines (*25*)*.* Because the upper part of gravity core DP30PC is disturbed during sampling, the dinoflagellate cyst temperature proxy was validated by comparing the cyst association composition of temperature-sensitive species of a well dated (^210^Pb/^137^Cs) multicore (*31*) collected near the study site (GeoB 10709-5; see supplementary text) to mean Italian late summer/autumn air temperatures (Fig. 2A). The strong positive correlation between our cyst record and autumn air temperatures confirms our proxy to reflect late-summer/autumn upper water conditions.

## RESULTS

**Fig. 3.**
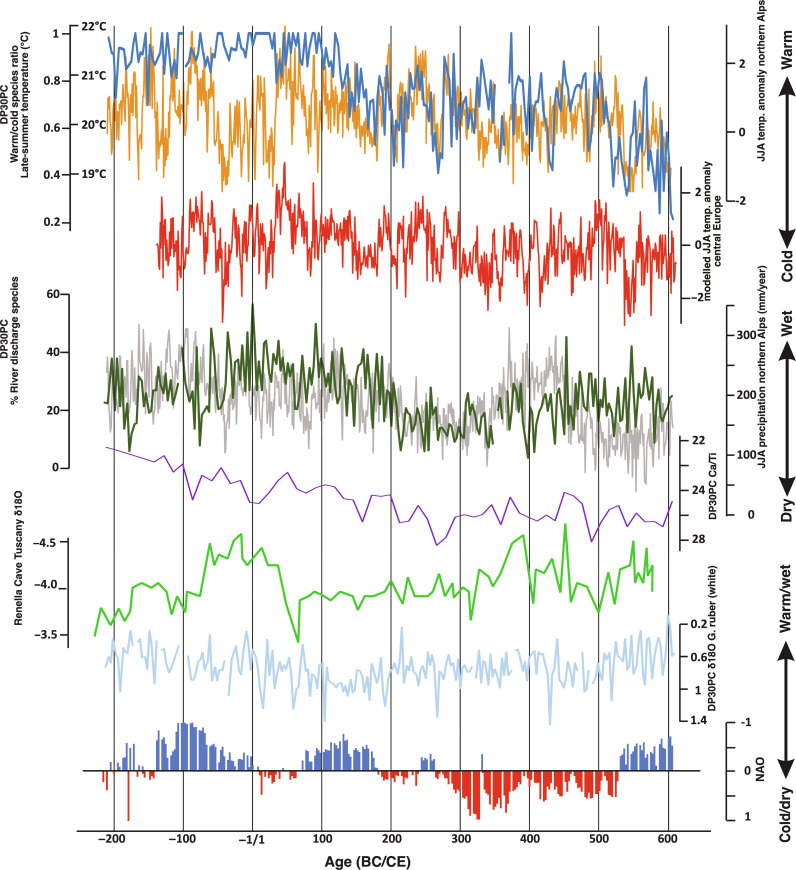
Core DP30PC and paleoclimatic records. Dark blue: Gulf of Taranto, late-summer temperature reconstruction based on dinoflagellate cyst composition (this study); orange: Northern Alps, June to August dendrochronological-based temperature reconstruction (*3*); red: proxy-based central European, June to August (JJA) temperature reconstruction (*33*); dark green: Core DP30PC Gulf of Taranto, late-summer river discharge (precipitation) reconstruction based on dinoflagellate cyst composition (this study); gray: Northern Alps, June to August dendrochronological-based precipitation reconstruction (*3*); purple: Gulf of Taranto, Ca/Ti concentrations (*73*); green: δ^18^O [per mil (‰) Vienna Pee Dee belemnite (VPDB)] of stalagmite RL4, Renella Cave Tuscany, Central Italy (*15*); light blue: DP30PC δ^18^O (‰ VPDB) of the planktonic foraminifera *Globigerinoides ruber* (white) (*76*) and NOA Index (*75*)*.*

The marine record documents four major phases in the temperature and precipitation history of ancient Roman Italy during the period covered by our study (Fig. 2B and data S1). The first phase (~200 BCE down to ~100 CE), in the beginning of our record, is characterized by persistently higher and relatively stable temperatures in southern Italy compared to later periods (Figs. 2B and 3). While the label of the Roman Warm Period or Roman Climate Optimum has been nearly ubiquitous in the paleoclimate literature, it is inconsistently deployed and so general as to be of limited use. Our record provides the first high-resolution confirmation of a generally warmer and stable period in the core region of the Roman Empire while also revealing short-term climate deviations. Prolonged warmth was notably evident in the first century CE, when the cyst association purely consisted of so-called “warm-water” species. The dinoflagellate cyst signal indicates relatively high amounts of discharge waters at the core location, suggesting that this warmer period was generally characterized by humid conditions in north-/eastern Italy (Figs. 2B and 3). Our record documents that this humid period was interrupted by episodes of short-term aridity in the early second century BCE and early first century BCE. The second phase (~100 CE to ~215 CE) is characterized by a decreasing trend of temperature and river discharge; the warmer temperature levels associated with the Roman Climate Optimum start to change as early as ~100 CE but more notably after ~130 CE when temperatures drop below the range of variation observed during the first phase. Pulses of cooler temperatures are observed between ~130 to ~145 CE, ~160 to ~180 CE, and ~200 to ~215 CE. This period is followed by a third phase (~215 to ~515 CE) in which southern Italian temperatures varied strongly. After a brief, somewhat warmer period from ~215 to ~245 CE, temperatures sharply declined, briefly reaching a low ~265 CE that lasted until ~275 CE and that would not be equaled again until ~518 CE. During this third phase, river discharge rates continued to decline, reaching their lowest levels across the entire study period during the second half of the third century CE, followed by a modest trend toward somewhat higher rates between ~350 to ~390 CE and ~410 to ~490 CE. These conditions changed in the early sixth century, when southern Italy witnessed a strong abrupt decrease of autumn temperatures with minimum values at ~537 and ~590 CE.

On the basis of existing literature, we assembled a comprehensive catalog of attested epidemic disease outbreaks in Italy across the eight centuries covered by our record (table S1). In our record, the three major pandemic events of Roman antiquity (*11*)—the Antonine Plague (onset ~165 CE), the Plague of Cyprian (onset ~251 CE), and Plague of Justinian (onset ~541 CE)—are strongly associated with pronounced climate change, although in varying ways. The Antonine Plague occurred during the cold pulse between ~160 and ~180 CE that followed several decades of trends toward cooling and aridity. The Plague of Cyprian coincides with a second phase of severe cooling, with even more arid conditions, after the brief warmer period between ~215 and ~245 CE. Last, the First Plague Pandemic originates at a moment of extreme cooling in the 530s to 540s, and recurrent outbreaks occur in the generally cooler sixth century, notably ~590 CE.

## DISCUSSION

Our climate reconstructions are generally in excellent agreement with high–temporal resolution climate reconstructions from northern Alps–based dendrochronology (Fig. 3) and proxy-based reconstructions of mean European summer temperatures (June to August, Fig. 3) even when considering an age uncertainty between 3.6 and 12 years [see Materials and Methods; (*3*, *14*, *32*)]. In line with these records, we observe that the episodes of large amounts of ASW at the core sites in the first part of our record (~200 BCE to ~100 CE) are interrupted by short spells of reduced ASW. These changes correspond to phases of aridity in the Alpine region and central Europe. This strong correlation is expected as the amount and maximal distal extension of the ASW plume in late summer/autumn is determined by precipitation in the Alps and northern/northwestern Apennines. These arid spells correspond not only to climate variability in the Alpine and central European region but also to phases when fewer floods of the Tiber are attested in historical records (*13*)*.* In agreement with the abovementioned alpine records, we observed moist conditions in the first century followed with a gradual decrease in precipitation in the first 350 years CE; then, from ~350 to 600 CE, our record reflects several phases of higher precipitation (Figs. 2B and 3). Similar trends in precipitation are observed in low–temporal resolution archives based on proxy data of Italian pollen records; speleothems archives from central Italy, Iberia, and central Mediterranean; as well as marine Mediterranean records and historical data (*13*, *15*, *21*, *33*–*36*)*.*

In contrast to the dendrochronology-based temperature reconstructions of the northern Alps, we observed persistently higher temperatures in southern Italy in the last two decades BCE and first century CE, underscoring the need for regionally specific proxies to understand the history of the Roman climate (Fig. 3) (*3*, *14*). This difference in temperatures between the northern Alps and southern Italy in the last two centuries BC and first century CE agrees with the well-documented high spatial variability in temperature history at different locations in the Mediterranean Sea region [e.g., (*13*, *37**–**40*)]. Although part of the discrepancy between these studies might be due to dating errors of several decades to centuries [e.g., see supplementary materials of (*39*)], in general, these low-resolution temperature reconstructions support our observation that the southern Italian region was characterized by warm stable temperatures in the first century CE followed by a cooling trend (*18*, *21*, *41*, *42*). Recently, it was suggested on the basis of earth system modeling that radiative forcing of the massive volcanic eruption of the Okmok volcano (Alaska) at 43 BCE caused a severe negative temperature anomaly in Italy during the 2 years following this eruption (*43*). Our record does not reflect anomalous cooling at this time, possibly because a temperature anomaly of 2 years is below the detection limit of our reconstruction.

Our data allow us to pinpoint the end of the stable conditions characterizing the Roman Climate Optimum already as early as ~100 CE and more notably after ~130 CE. From this time on, temperature fluctuations move more or less in line with temperature fluctuations in northern Europe and the northern Alps (Fig. 3) with short spells of warmer conditions. Conditions changed abruptly after ~515 CE, when southern Italian temperatures decreased synchronously with the dendrochronological record of the Alps and mean European temperature reconstructions to show a strong minimum at 537 CE. Our record confirms the sharp onset and severity of the Late Antique Little Ice Age in southern Italy, with reconstructed temperatures in the final part of the sixth century about 3° colder than the highest decadal averages during the Roman Climate Optimum. Multi-proxy reconstructions have indicated the beginning of an anomalously cold period in 536 CE (*14*)*.* A prolonged episode of solar veiling is widely attested in written records, and volcanic tephra indicate a northern-hemisphere eruption, the first of at least three large eruptions in the succeeding decade (*44*, *45*)*.* According to reconstructions based on dendrochronological records from the Altai Mountains and the Alps, the 540s CE were the coldest and second-coldest decades, respectively, in the Common Era (*14*)*.* While the extent and duration of cooling have been questioned (*46*), as have the social effects of sixth-century climate change (*47*), our record underscores the magnitude, abruptness, and longevity of changed climate conditions from ~537 CE in this core region.

Our reconstruction opens the possibility of exploring the complex interplay of natural and human systems in the heartland of the ancient Roman Empire at a high level of chronological resolution and regional specificity. The full causal implications of the changing climate for agricultural systems, demographic patterns, and social impact in Roman Italy will require fuller investigation, but immediately notable associations include (i) general confirmation of a warmer, humid, and stable phase during the expansionary period of the late Roman Republic and early Roman Empire; (ii) the early if gradual termination of this climate pattern from ~100 CE and more pronounced from ~130 CE; (iii) prolonged environmental variability across the later Roman imperial period; and (iv) regional evidence for the likely significance of the Late Antique Little Ice Age. Above all, our reconstruction suggests an association between phases of climate change and episodes of acute health crisis.

**Fig. 4.**
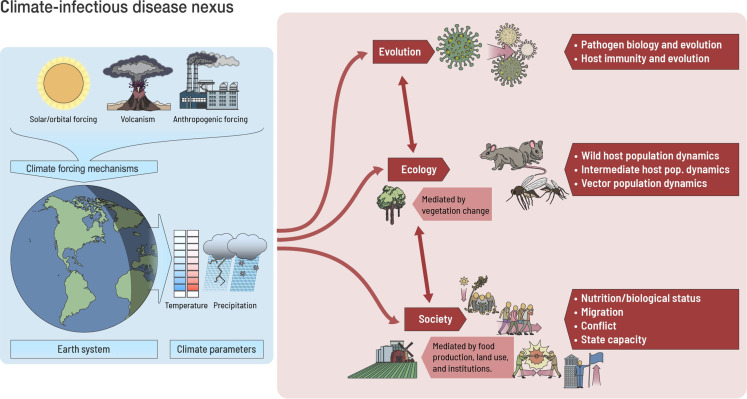
Schematic drawing of the relationship between climatic change and sociological, physical, and biological factors influencing infectious disease outbreaks.

The mechanisms linking physical climate change and infectious disease are notably complex (*10*), and, in our case, only some possibilities can be briefly suggested for consideration and future investigation (Fig. 4). Epidemic disease outbreaks always involve a mix of social (migration, nutritional status, and state capacity), ecological (wild host, intermediate host, and vector population dynamics), and evolutionary (pathogen physiology and adaptation) factors (*9*, *48*)*.* Climate-induced stress could have acted as a trigger or amplifier of a disease outbreak, playing a role in the onset or intensification of epidemic mortality (or both). For instance, climate stress might trigger outbreaks by contributing to spillover events as animal host and vector populations change, by weakening a society’s ability to respond to crisis (*49*), by spurring migration and conflict (*50*), or by causing biological stress that renders a population vulnerable to further health insults (*51*)*.*

Studies on late medieval and early modern Italy have shown that climate-induced food shortage likely played a role in catalyzing or exacerbating mass mortality events (*52*)*.* It has been suggested that, in early modern Italy, episodes of short-term climate instability, especially rapid cooling, were associated with food shortage, social crisis, and epidemic disease (*6*, *53*)*.* It can therefore be assumed that this might hold for Roman times as well. Agriculture in Italy is responsive to variation in the timing and magnitude of both seasonal rainfall and temperature, and, in general, yields in southern Italy are more responsive to precipitation, while those in the north are more temperature-sensitive (*54*, *55*)*.* These relationships are nonlinear, with extremes in either direction of temperature or rainfall threatening to reduce yields (*56*)*.* Much of Italy is hilly, and elevation changes between the Apennine uplands and coastal lowlands ensure local variation and differing responses to short-term environmental stimuli (*57*)*.* Within the ranges of variation indicated by our record, it is likely that warmer and more humid conditions were conducive to superior agricultural output, while cooler and drier conditions depressed productivity and increased the chance of catastrophic harvest failure. However, more research is needed to investigate these relationships in the dynamics of ancient agriculture by taking into account the lower yields and longer time to crop maturity that are suggested for this time period.

Understanding the mechanistic links between climate and disease in our case is even more complicated by the fact that the three major outbreaks occurring in the studied time interval were true pandemics. These interregional events were not confined to Italy, and our climate record should ultimately be combined with others for a full understanding. With respect to these severe outbreaks, we assume that various mechanisms were at work simultaneously. The Antonine Plague follows a few decades of cooling and increasing aridity, during what is generally believed to be a time of population expansion and perhaps pressure in parts of Italy (*58*)*.* There has been disagreement over whether climate change played a role in the onset of the pandemic, with some emphasizing its role (*59*, *60*) and others being more skeptical (*11*)*.* Still others focus on the likely role of climate change in exacerbating the crisis (*61*). With the pathogenic agent and geographic origin of the outbreak unknown, there will remain uncertainty. However, our core sheds light on conditions in Italy during the decades preceding and overlapping the crisis. The marine core–based reconstruction is consistent with the view that several decades of climate-influenced stress on the peninsula may have set the conditions for pandemic mortality, which was then exacerbated by simultaneous abrupt climate change (*60*, *61*)*.*

A second phase of severe cooling with even more arid conditions occurred after the brief warmer period between ~215 and ~245 CE, overlapping the so-called Crisis of the Third Century (~250 to 275), a period of exceptional political turmoil characterized by monetary crisis, imperial fragmentation, and pestilence (the Plague of Cyprian) (*62*)*.* Our record lends strong support to the inference that climate change was a substantial stressor in Italy during this multifaceted crisis. The second half of the third century witnessed severe settlement contraction in Italy (*63*)*.*

Last, the strong cooling of the Late Antique Little Ice Age in southern Italy coincided with the onset of the First Plague Pandemic, a disease outbreak caused by the bacterium *Yersinia pestis*, the biological agent of the medieval Black Death (*48*, *64*), and it is associated in many regions globally with a phase of crisis (*65*)*.* The full relationship between the onset of the Late Antique Little Ice Age and the First Plague Pandemic has prompted discussion and speculation without resulting in confident conclusions (*60*), and the sharp cooling could well have affected food production, animal host populations, and human social changes all at the same time. The pandemic entered the Roman Empire across its southeastern frontiers, via the Red Sea and upper Egypt (*66*), although the location of the immediate animal host population is unknown. The plague reached Italy by 543 CE (*67*), and our record supports the hypothesis that local climate conditions would have played a role in amplifying the effects of the disease event. Recurrent plague outbreaks in the cooler sixth and seventh century (in concert with persistent warfare and other factors) drove the Italian population to a nadir in the second half of the sixth or seventh century CE (*68*).

While not all episodes of cooling in our record are clearly linked with known outbreaks of epidemic mortality (see the downturn at ~200 to 215 CE), the association now documented by the marine core–based reconstruction underscores that climate change on the order of 1° to 3°C on decadal timescales substantially stressed ancient societies and increased susceptibility to major health impacts.

Modern societies have many resources to withstand environmental shocks that were unavailable in ancient times, such as high-yield agriculture, global trade networks, and biomedical science. Nevertheless, the COVID-19 pandemic underscored the continuing risk represented by emerging and reemerging infectious diseases. Past episodes of climate change can provide a resource for understanding the complex links between environmental challenge and social resilience. Given the rarity of the largest-magnitude epidemic events, which likely involve a concatenation of environmental and social factors, long timescales are necessary for a fuller understanding of the true risks of climate change. The marine core–based reconstruction presented here underscores the systemic importance of the climate system in shaping human health and social stability. The health impacts of climate change, including the threat of emerging infectious diseases, are increasingly recognized [Intergovernmental Panel on Climate Change (IPCC; 2022)]. However, it is inherently difficult to measure the risks posed by rare outlier events such as those that are triggered by contagious diseases (*69*–*72*)*.* Nonetheless, our record emphasizes that climate change, pandemic disease, and the fate of human societies have been linked for thousands of years and that such links should be considered in evaluations of future risks to global well-being and stability.

## MATERIALS AND METHODS

### Experimental design

We reconstructed Italian temperature and precipitation history from ~200 BCE to ~600 CE based on the dinoflagellate cysts association in sediments of the marine archive DP30PC from the Gulf of Taranto and compared these with the occurrences of epidemics and pandemics in Italy. The calibration of our dinoflagellate cyst–based temperature proxy is based on the comparison of the cyst association of a reference dataset from multicore core GeoB 10709-5 (*29*) to mean Italian late-summer/autumn air temperatures (*32*).

### Material

Gravity Core DP30PC (at 39°50.07 N, 17°48.05 E, water depth of 270 m) and Multicore GeoB 10709-5 (39°45.39 N, 17°53.57 E, water depth of 172 m) were collected during the RV Pelagia cruise “DOPPIO” in 2008 and RV Poseidon cruise “CAPPUCCINO” in 2006, respectively (*73*)*.* Investigations have been carried out in DP30PC core section 8. Lithology of cores DP30PC section 8 and GeoB 10709-5 are formed by homogeneous green-gray sediments of uniform mud/clay size fraction (fig. S1) (*74*).

#### Tephrochronology and age model of core DP30PC

The age model of DP30PC is based on tephrochronology complemented by ^14^C and ^210^Pb/^137^Cs-datings. For tephrochronological measurements, the glass shard contents in % noncalcareous mineral particles of mineral size fraction between 100 and 20 μm have been determined for the core-depth interval between 849 and 1629 mm (table S2). For this, the core was sliced in slices of 2.5 mm. About 0.1-g dry weight of material was treated with 10% HCl to remove carbonates. To remove particles larger than 100 μm, the material was sieved over a metal sieve with pore size of 100 μm. Successively material was sieved over a high precision sieve (Strock-Veco) with pore size of 20 μm. The residual material was moved to an object glass, embedded in glycerine gelatine, and sealed with parafine wax. The number of volcanic particles relative to nonvolcanic silicate particles was determined by polarized light microscopy (fig. S1).

From selected intervals with enhanced volcanic glass concentrations (cryptotephra), additional material was treated with 10% HCl to remove carbonates. Successively, the material was sieved over metal sieves of 100 and 45 μm. Single volcanic glass shards of the 45- to 100-μm particle size fraction were selected by hand and were embedded in 2.54-cm mounts, using Buehler EpoThin epoxy resin. The mounts were ground to 5 to 9 μm until sufficient glass shards were exposed and then polished on a cloth with diamond suspension to 0.25 μm. Major element compositions were determined on the carbon-coated mounts by a Cameca SX-100 electron probe microanalyzer at the Faculty of Geosciences, University of Bremen. Analytical conditions included an acceleration voltage of 15 kV, beam current of 3 nA, and a defocussed beam of 5-μm diameter. Counting times were 20 s on peak and 10 s on each background position. Sodium was the first element to be analyzed, and it was verified on rhyolite glass that no Na loss occurred during the analyses. The instrument was calibrated with Smithsonian reference materials VG-2 glass (Al, Si, Mg, Ca, and Fe), ilmenite (Ti and Mn), anorthoclase (Na), microcline (K), and fluorapatite (P), using the values of Jarosewich *et al*. (*75*)*.* The data were standard-corrected using the reference material VG-A568 (rhyolite glass) analyzed multiple times during each analytical session. Analytical quality was monitored by analyses of the reference material VG-A99 (basalt glass) along with the samples; accuracy and precision are summarized in table S3. Elemental composition of individual volcanic glass particles is given in data S2.

Three ^14^C-datings based on mixed planktonic foraminifera samples have been determined in the upper part of the core (table S4) (*73*, *76*). Comparison of 7 ^210^Pb measurements in the upper part of core DP30PC with 15 measurements in the upper 25 cm of a nearby multicore NU 04 as well as the ^14^C datings of both cores revealed that the top sediments of core DP30PC represented sediments of about 10 years younger than the recovery date representing the year 1998 (*74*, *77*)*.* It also suggests that sediments in the upper ~10 cm of core DP30PC are bioturbated.

In the studied core section, four distinct layers with glass shards could be distinguished (fig. S1 and table S2). Comparison of the chemical composition of the isolated particles to that of glass particles isolated from tephras and cryptotephras in eastern Mediterranean sediment archives (*78*–*86*) allowed the linking of three of the cryptotephras to known volcanic outbreaks.

Cryptotephra 1 (1601.5- to 1579-mm core depth, 79 CE): The first increases of volcanic glass particles are observed between 1601.5- and 1579-mm core depth with maxima documented at 1596.5- and 1591.5-mm core depth. When only the ^14^C dating points (second polynome age model) are taken into consideration, this cryptotephra has a deposition time between 81 and 113 years CE ± 120 years. The majority of the glass particles at 1596.5- and 1594-mm core depths have a clear Lipari composition (*80*, *82*)*.* The elemental compositions of the “Lipari” glass shards are equivalent to that of glass particles in cryptotephra “t6” of the core TEA-C6 from the western side of the Gulf of Taranto (fig. S2 and data S1) (*82*). In this latter core, particles with a Lipari signal were mixed with glass particles that could be assigned to the Somma-Vesuvius outbreak at 79 CE. This allows us to assign cryptotephra 1 observed in this study as cryptotephra t6 of core TEA-C6.

Within cryptotephra 1, we observed only one glass shards with a composition that is equivalent of both the Somma-Vesuvius and Mt. Etna outbreaks (1596.5-mm core depth; fig. S1 and data S2). No indication is present that the Mt. Etna erupted at this time. We therefore assume this glass shard to originate from the Somma-Vesuvius. The ash-particle fallout region of the Somma-Vesuvius 79 CE outbreak is thought to have had its maximum extent west of the core position of DP30PC, and it can thus be expected that Somma-Vesuvius shards are rare in core DP30PC (*83*)*.*

Our findings are in contrast with previous studies that ascribed enhanced amounts of pyroxene particles in a core collected at the same position as DP30PC to the Somma-Vesuvius 79 CE outbreak (*87*, *88*). However, in these studies, the elemental signal of glass particles was not identified and as such could not be related to Lipari volcanoes. Furthermore, at the time of publication of these studies, it was not known that Lipari ash had reached the Gulf of Taranto as early as 79 CE. It is therefore not clear whether the pyroxene particles documented in these studies originated from the Somma-Vesuvius.

Cryptotephra 2 (1329- to 1279-mm core depth): After a barren interval, a second interval with increased numbers of volcanic glass particles is observed, which, according to the ^14^C dating points, would represent the time interval between 463 and 532 years CE ± 120 years. A similar observation was found in core TEA-C6 where, after a quiescent period, additional cryptotephras were present whose ages were estimated to be about 437 CE without providing information about the age uncertainty (*89*)*.* Glass particles from this cryptotephra in TEA-C6 had chemical compositions characteristic for both Lipari and Mt. Etna outbreaks, suggesting a time equivalent outbreak of both volcanoes. The glass particles analyzed from DP30PC showed only a Lipari composition (fig. S2 and data S2), suggesting that fallout of the Mt. Etna did not reach the core site. Unfortunately, no exact dating can be assigned to this cryptotephra based on the elemental analyses of their glass shards.

Cryptotephra 3 (1259- to 1244-mm core depth, 512 CE): The interval between 1259- and 1244-mm core depth (^14^C-based age: 559 to 580 CE ± 120 years) contains glass shards that have either a Lipari or a Somma-Vesuvius composition. The latter have chemical compositions similar to the TM–2a Tephra in the nearby lake Lago di Monticchio (*90*)*.* This tephra can be assigned to the historical 512 CE eruption (fig. S2 and data S2) that is associated to the late phase of the “Pollena eruptive cycle” between 472 and 512 (*83*, *91*)*.* We therefore assign the recording of the base of this cryptotephra at 1259 mm to represent the year 512 CE.

Cryptotephra 4 (1079- to 1059-mm core depth, 776 CE): After a quiet interval, an additional cryptotephra with a clear Lipari composition was observed (^14^C-based age: 751 to 833 CE ± 120 years). The chemical similarity of the individual Lipari outbreak phases does not allow an unequivocal separation between them. Nevertheless, the position in the core and the similarity in chemical composition of glass shards observed in core GeoB 15403-3 that is cored in very close vicinity of core DP30PC suggest that these glass shards represent ash particles of the 780 CE Monte Pilato outbreak (fig. S2) (*80*)*.* This is in agreement with observations that the plume of the Monte Pilato outbreak passed the core location (*81*)*.* Therefore, we assume the analyzed glass shards at 1066.5-mm core depth to be deposited at 780 CE.

On the basis of these three dating points, 79, 512, and 780 CE and assuming the top of the core representing the years 1998 CE, the following age-depth relationship could be established on the basis of a second polynome model that compensates for compaction in the upper part of the core

$$y=-1.0036037\times 10^{-4}x^{2}-1.044337x+1998.5$$

$$R^{2}=0.99987$$

where *y* is the age (years BCE/CE) and *x* is the core depth (millimeters).

On the basis of this age model, cryptotephra 2 is deposited between 433 and 499 CE (fig. S3). According to this model, the deposition age of sediment at the ^14^C measurement points represents the years 187 BCE and 1112 CE that are in range with the ^14^C dating results (fig. S3 and table S5). The polynome-based age differs from the absolute age at the dating points by 3.6 years (cryptotephra 1), 12 years (cryptotephra 3), and 9 years (cryptotephra 4). On the basis of the homogenous character of the lithology and size fraction in the studied core section, no indication is found of varying sedimentation rates other than caused by compaction of the core sediments. Therefore, we have no indication that a variation in age uncertainty between the dating points exceeds that of those at the dating points. We therefore assume that the age uncertainty varies from 3.6 years in the lower part of the core toward 12 years in the upper part of the core.

#### Temperature and precipitation reconstructions based on dinoflagellate cysts

Qualitative information regarding variations in autumn sea surface temperature was obtained using the following ratio

$$W/C=W_{n}/\left(W_{n}+C_{n}\right)$$

where *n* represents the number of specimens counted; *W* represents the warm-water species: *Impagidinium aculeatum*, *Impagidinium patulum*, *Impagidinium paradoxum*, *Operculodinium israelianum*, *Polysshaeridium zoharyi*, and Spiniferites mirabilis; and *C* represents the cold-water species: *Spiniferites elongatus* and *Bitectatodinium tepikiense.*

Qualitative information regarding variations in autumn upper water nutrient concentrations reflecting river discharge

$$\text{DI}={\text{ASW}}_{n}/\left({\text{ASW}}_{n}+R_{n}\right)$$

where *DI* is the discharge index; ASW*_n_* is the total counts of specimens of dinoflagellate cysts and fresh-water algae characteristically present in ASW (*92*): *Lingulodinium polyedrum*, cysts reworked from pre-Holocene sediments, the freshwater algae *Pseudoschizea* spp.; and *R_n_* is the total counts of specimens of dinoflagellate cyst species resistant to postdepositional aerobic degradation (*93*): *Ataxiodinium choanum*, *B. tepikiense*, *I. aculeatum*, *I. paradoxum*, *I. patulum*, *Impagidinium plicatum*, *Impagidinium sphaericum*, *Impagidinium strialatum*, *Nematosphaeropsis labyrinthus*, *O. israelianum*, *Pyxidinopsis reticulatum*, *Polysphaeridium zoharyi*, *S. elongatus*, *S. mirabilis*, *Spiniferites ramosus*, and *Tectatodinium pellitum.*

Species preference for the abovementioned upper water environmental parameters is based on the present-day distributions of cysts in surface sediments in the research area as well as their global distributions (*92*–*94*)*.* To avoid potential disturbance by postdepositional species selective degradation, only species resisting aerobic degradation have been taken into account (*95*)*.*

The dinoflagellate *W*/*C* ratio of samples from core GeoB 10709-5 has been compared to instrumental late-summer mean Italian temperatures between 1774 and 2014 at an altitude of ~1000 m (Fig. 2A (*32*)*.* Comparison between this compilation and instrumental data from the cities Rome (between 1811 and 1985), Taranto (between 1951 and 1967), Brindisi (between 1951 and 1985), and Foggia (between 1811 and 1985) derived from “climate explorer” service of the Royal Dutch Meteorological Survey [Koninklijk Nederlands Meteorologisch Instituut; http://climexp.knmi.nl, monthly station data, GHCN-M (adjusted), mean temperature, stations with name containing: Rome/Taranto/Brindisi/Foggia] indicates that, for the time interval between 1951 and 1967 (overlapping time interval of all instrumental records), air temperatures at sea level are 8.4°C warmer than at an altitude of 1000 m. For establishing a transfer function, the *W*/*C* data have been transformed to annual values using the program Past V4.02 (*96*). A regression between the three-point mean of both the *W*/*C* curve and the mean Italian instrumental data of Leonelli adapted for sea level temperatures was established. This results in the following relationship between the *W*/*C* dinoflagellate cyst ratio and instrumental mean Italian air temperature data adapted for sea level temperatures

y=(4.7278x+17.03)

(R=0.44)

where *y* is the calculated temperature in degrees Celsius and *x* is the *W*/*C* ratio.

Unfortunately, no correlation between the *DI* and instrumental data could be established as no instrumental river discharge data are available for the time period before the widespread use of artificial fertilizer in Europe and pollution of the Italian rivers by nitrogen and phosphate (*29*)*.*

#### Catalog of epidemic mortality in Roman times

The period covered by our reconstruction encompasses the late Roman republic, the early Roman Empire, and the later Roman Empire (or late antiquity). For most of this period, Rome was the primary seat of a sprawling territorial empire that at its apex encompassed ~5,000,000 km^2^ and stretched across more than 33° latitude north to south and 34° longitude east to west (*97*)*.*

Drawing on existing secondary literature (*67*, *98*–*103*), the dataset presented in table S1 is a comprehensive catalog of known epidemic mortality events attested in Italy during this period. Several limitations of this dataset need to be emphasized, starting with its heterogeneity. Our knowledge of past episodes of epidemic disease derives from literary sources such as ancient histories, not from standardized government or ecclesiastical records. Moreover, ancient cultures mostly lacked modern concepts of germ theory, which allow the conceptual linkage between microbiological agents and specific diseases (*101*, *102*)*.* Hence, the ancient terminology for disease outbreaks was highly generic; in Latin, the most common terms were pestis, lues, pestilentia, mortalitas, morbus, and clades (*103*)*.* Moreover, ancient reports of epidemics were often highly rhetorical, their interpretation colored by political and social concerns (*104*)*.* The catalog of epidemic events offered below can be no better than the underlying record, imperfect as it is, allows. However, note that the three pandemic events “The Antonine Plague” (~165 to 180 CE), the “Cyprian Plague” (251 to 266 CE), and the “First Plague Pandemic” (541 to 766 CE) are exceptionally well attested in the historical record by a variety of contemporary observers. Despite the inherent limitations of such a record, it is worth exploring the potential links between climate change and attested outbreaks.
