# Supplementary material for: Climate change, society, and pandemic disease in Roman Italy between 200 BCE and 600 CE
Source: Sci Adv. 2024 Jan 26;10(4):eadk1033. doi: 10.1126/sciadv.adk1033 (PMC10816712; doi:10.1126/sciadv.adk1033)
Supplement: Supplementary file 1 — Supplementary text Figs. S1 to S3 Tables S1 to S5 Legends for data S1 to S3 [file sciadv.adk1033_sm.pdf]

Science Advances
AAAS

Supplementary Materials for

Climate change, society, and pandemic disease in Roman Italy between 200 BCE and 600 CE

Karin A. F. Zonneveld *et al.*

Corresponding author: Karin A. F. Zonneveld, kzonneveld@marum.de

Sci. Adv. **10**, eadk1033 (2024)
DOI: 10.1126/sciadv.adk1033

The PDF file includes:

Supplementary text
Figs. S1 to S3
Tables S1 to S5
Legends for data S1 to S3

Other Supplementary Material for this manuscript includes the following:

Data S1 to S3

Supplementary text

Verification of the dinoflagellate cyst proxy

The dinoflagellate cyst proxy was verified by comparing the cyst association composition of temperature-sensitive species of the multicore GeoB 10709-5 that was collected near the study site *(29)* to mean Italian late summer/autumn air temperatures *(32)*. Prior to correlation, an age model of this core has been established that takes into account the rate of sediment compaction.

The age assessment of core GeoB 10709-5 is based on ^{210}Pb /^{137}C -dating by gamma spectroscopy at the Institute of Environmental Physics, University of Bremen (table S5). Based on the methodology described in *(29),* wet samples were sealed into plastic cylindrical dishes with a diameter of 7 cm using Rn tight foil. Before measurement they were left sealed for a minimum of 3 weeks so that the radioactive equilibrium between ^{226}Ra and ^{222}Rn (and its daughters) had been established. A coaxial HPGe detector Canberra Industries (50% rel. efficiency) housed in a 10 cm Pb shielding with Cu and plastic lining operated under Genie 2000 software was used for gamma spectroscopy. Measurement live-times were 2 days or more. The full energy peak efficiencies have been calculated using LabSOCS© (Laboratory Sourceless Calibration System), Genie 2000 software calibration tool, based on defined sample to detector geometry and density. For determination of excess-^{210}Pb activity ($^{210}Pb_{xs}$) ^{210}Pb -supported activity was subtracted from the ^{210}Pb -total signal, measured via 46.5 keV line. Supported ^{210}Pb was determined via the 351.9 keV line of ^{214}Pb. Additionally, the artificial isotope ^{137}C was analyzed. ^{137}C values above the limit of detection were found in the uppermost 6 slices (down to depth of 12 cm). $^{210}Pb_{xs}$ decreases monotonously in the profile with maximum value of 106 Bq/kg in the depth of 0.5cm.

To determine the rate of compaction in the core, dry bulk densities (DBD, g/cm^3) have been determined every 0.25 cm. DBD has been determined by drying 1 cm^3 material overnight at 60°C directly after slicing the core. Successively the dry weight was determined using a high precision weight. Unfortunately, at several core depths, material was lost during the slicing process of the upper 9 cm of the core due to the large water content. As such reliable DBD values could only be determined for the depth intervals between 3 - 4 cm and between 9 - 40 cm (Data S3). The relationship between core depth and DBD has been calculated with the program Past V4.02 *(97).* Based on this relationship modeled DBD values have been calculated according to the equation:
$y = 0.21923x^{0.28918}$
where y= modeled DBD, x = core depth (mm)

The compaction of the core has been calculated with the program Past V4.02 using an exponential model. With this model compaction-corrected analysis, modelled depths have been calculated for the ^{210}Pb measurements with the following equation:

$y = 35.743e^{(1.658x)}$
where y = compaction corrected depth (mm) and x = modeled DBD (g/cm^3)

Sedimentation rates have been determined based on the compaction adapted core depths using the ^{210}Pb data excluding the uppermost point (assuming a bioturbation depth of 5 cm). For this an

exponential model has determined with the program Past V4.02 using a half-life time of ^{210}Pb of 22.2 years with the following equation:

Dinoflagellate cyst processing

For dinoflagellate cyst analyses, sediment samples were dried at 60°C, weighed and treated with 10% HCl and 40% HF in subsequent steps to dissolve calcium carbonate and silicate. Decantation was performed by sieving over a 20 µm high precision sieve (Storck-Veco) to prevent loss of material. The residues were transferred to an Eppendorff tube, centrifuged for 8 min at 3000 rpm after the material was concentrated to a volume of 1 ml. This material was homogenized, after which subsamples of a known volume (50–100 ml) were placed on a microscope slide, embedded in glycerin jelly and sealed with paraffin wax. Whole slides were counted for dinoflagellate cysts using a light microscope at 400x magnification. When the slides contained less than 100 specimens, additional slides were counted. Count data of core DP30PC and GeoB 10709-5 are given in Data S1 and S3 respectively.

$y = 928.11^{(-0.020388x)} - 8.4199$

where y = ^{210}Pb value and x = compaction adapted core depth (mm).

This results in a sedimentation rate of 1.3835 compaction corrected mm/year (Data S3). ^{137}Cs values above the limit of detection were found in the uppermost 12 cm. The onset of the nuclear fallout isotope ^{137}Cs in the core then would be expected at the depth of 6.5 cm (around year 1955), according to this model. However, penetration of the isotope into deeper layers due to continuous mixing at the top of the core can cause appearance of ^{137}Cs down to greater depth (approx. 13 cm). The age model derived from ^{210}Pb is therefore not in contradiction with ^{137}Cs data.

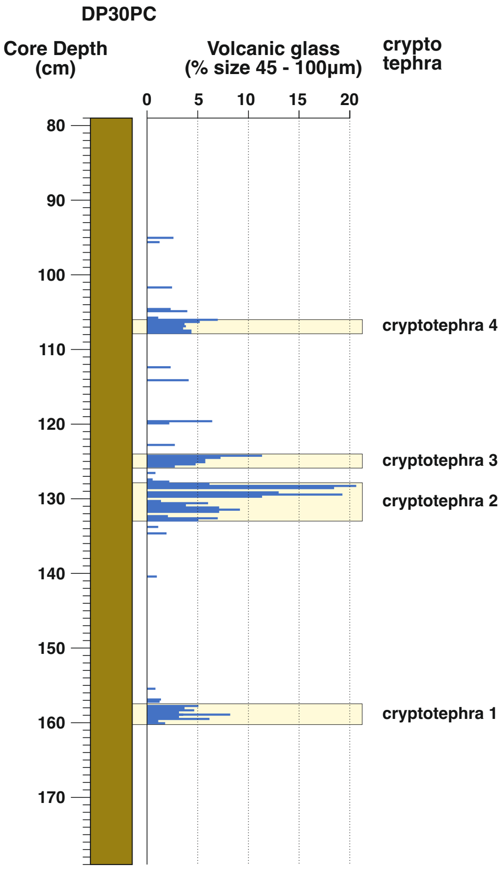

Fig. S1.

Core DP30PC glass shard concentration in the size fraction of 45 - 100 µm and positions of cryptotephras

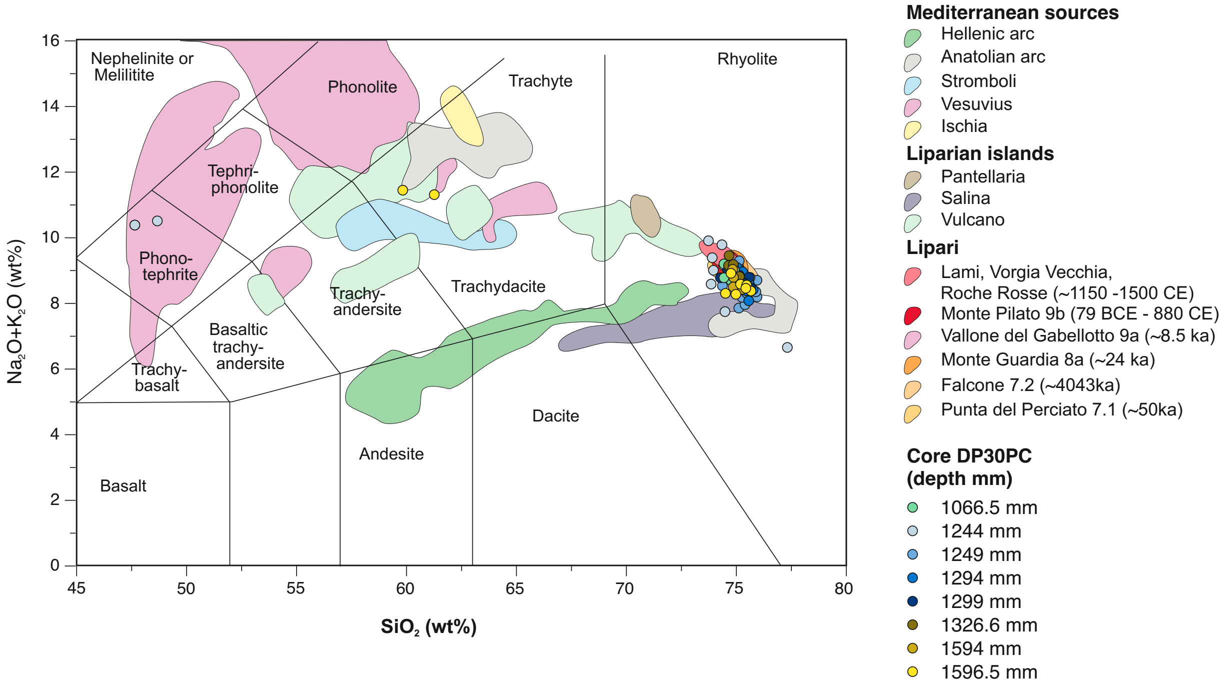

Fig. S2.

Summary of analyses of reference materials VG-A568 (rhyolite glass) and VG-A99 (basalt glass).

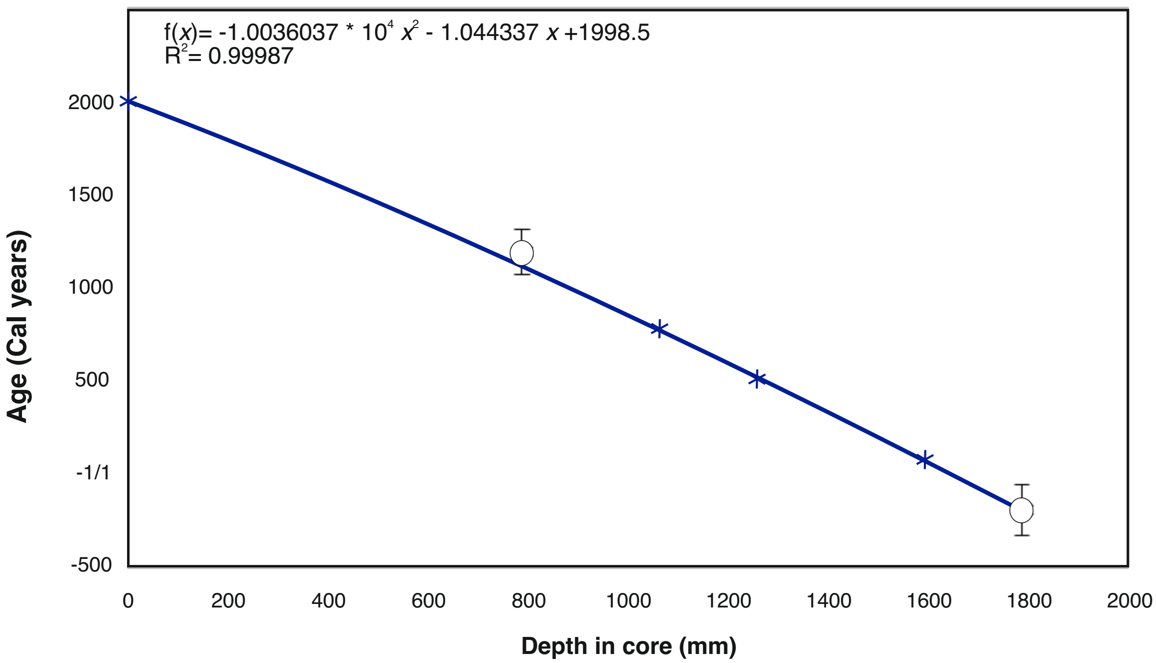

Fig. S3.

Age depth model of core DP30PC. Solid dots: dating points, Open circles: position of the ^{14}C measurements.

Age (years BCE/CE)	Source of recording
-187	Livy 38.44.7
-181	Livy 40.19.3; Julius Obsequens 6
-180	Livy 40.36.14, 40.42.6;
-174	Livy 41.211.5-11; Julius Obsequens 10; Ovid Fasti 5.315-20
-165	Julius Obsequens 13
-142	Julius Obsequens 22
-43	Dio 45.17.8
-23	Dio 53.33.4
-22	Dio 54.1.3
65	Tacitus Annales 16.13; Suetonius Nero 39; Orosius Historiae Adversus Paganos 7.7.10-11
77	Orosius Historiae Adversus Paganos 7.9
79	Suetonius Titus 8.3; Epitome de Caesaribus 10.13; Jerome Chronicon 65; Dio 66.23.5
80	Suetonius Titus 8.3; Epitome de Caesaribus 10.13; Jerome Chronicon 65; Dio 66.23.5
166-180	Antonine Plague (numerous: see Elliott 2024)
168	Galen In Hippocratis librum iii epidemiarum commentarii IV 1.29
191	Dio 73.14.3
251-266	Cyprian Plague (numerous: see Harper 2015; Harper 2016)
262	Scriptores Historiae Augustae Gallieni duo 5.5
397	Stathakopoulos no. 37
398	Stathakopoulos no. 37
408	Stathakopoulos no. 42
451	Stathakopoulos no. 65
452	Stathakopoulos no. 65
467	Stathakopoulos no. 70
471	Stathakopoulos no. 72
537	Stathakopoulos no. 94
539	Stathakopoulos no. 97 + 100
543-766	Justinianic Plague (numerous: see Stathakopolous 2004; Harper 2017; Harper 2023a)
554	Harper 2023a; poss 555 too
561	Harper 2023a; poss 562 too
565	Harper 2023a
571	Harper 2023a
588	Harper 2023a
590	Harper 2023a
591	Harper 2023a
592	Harper 2023a
599	Harper 2023a
600	Harper 2023a

Table S1.

Table of epidemic outbreaks in Roman Italy *(67, 99-104)*

Core DP30PC

Core depth (mm)	volcanic glass shards (% particles size fraction 45-100 µm)	Core depth (mm)	volcanic glass shards (% particles size fraction 45-100 µm)	Core depth (mm)	volcanic glass shards (% particles size fraction 45-100 µm)	Core depth (mm)	volcanic glass shards (% particles size fraction 45-100 µm)	Core depth (mm)	volcanic glass shards (% particles size fraction 45-100 µm)	Core depth (mm)	volcanic glass shards (% particles size fraction 45-100 µm)	Core depth (mm)	volcanic glass shards (% particles size fraction 45-100 µm)
844	0	1026.5	0	1096.5	0	1279	2	1461.5	0	1531.5	0	1601.5	1.64
846.5	0	1029	0	1099	0	1281.5	5.5	1464	0	1534	0	1604	0
849	0	1031.5	0	1101.5	0	1284	18.6	1466.5	0	1536.5	0	1606.5	0
851.5	0	1034	0	1104	0	1286.5	16.67	1469	0	1539	0	1609	0
854	0	1036.5	0	1106.5	0	1289	0	1471.5	0	1541.5	0	1611.5	0
856.5	0	1039	0	1109	0	1291.5	7.32	1474	0	1544	0	1614	0
859	0	1041.5	0	1111.5	0	1294	11.69	1476.5	0	1546.5	0	1616.5	0
861.5	0	1044	0	1114	0	1296.5	17.39	1479	0	1549	0	1619	0
864	0	1046.5	2.04	1116.5	0	1299	10.26	1481.5	0	1551.5	0	1621.5	0
866.5	0	1049	3.61	1119	0	1301.5	0	1484	0	1554	0	1624	0
869	0	1051.5	0	1121.5	0	1304	1.23	1486.5	0	1556.5	0.78	1626.5	0
871.5	0	1054	0	1124	0	1306.5	5.48	1489	0	1559	0	1629	0
874	0	1056.5	0	1126.5	2.15	1309	3.08	1491.5	0	1561.5	0		
876.5	0	1059	0.96	1129	0	1311.5	3.45	1494	0	1564	0		
879	0	1061.5	6.33	1131.5	0	1314	6.4	1496.5	0	1566.5	0		
881.5	0	1064	4.72	1134	0	1316.5	8.26	1499	0	1569	1.23		
884	0	1066.5	3.33	1136.5	0	1319	6.45	1501.5	0	1571.5	0		
886.5	0	1069	0.97	1139	0	1321.5		1504	0	1574	1.15		
889	0	1071.5	3.45	1141.5	3.7	1324	1.83	1506.5	0	1576.5	0		
891.5	0	1074	3.23	1144	0	1326.5	6.25	1509	0	1579	4.55		
894	0	1076.5	4	1146.5	0	1329	4.55	1511.5	0	1581.5	3.28		
896.5	0	1079	4	1149	0	1331.5	0	1514	0	1584	4.17		
899	0	1081.5	0	1151.5	0	1334	0	1516.5	0	1586.5	2.78		
901.5	0	1084	0	1154	0	1336.5	0	1519	0	1589	3.26		
904	0	1086.5	0	1156.5	0	1339	1.01	1521.5	0	1591.5	7.45		
906.5	0	1089	0	1159	0	1341.5	0	1524	0	1594	2.88		
909	0	1091.5	0	1161.5	0	1344	0	1526.5	0	1596.5	5.5		
911.5	0	1094	0	1164	0	1346.5	0	1529	0	1599	1.02		

Table S2. Gravity core DP30PC counts of volcanic glass shards in core section 8

Summary of standard analyses.

	SiO_2	TiO_2	Al_2O_3	FeO	MnO	MgO	CaO	Na_2O	K_2O	P_2O_5	Total
VG-A99 (N=9)											
Average (wt%)	51.20	4.06	12.50	13.52	0.19	5.04	9.28	2.67	0.83	0.42	99.71
RSD%	0.7	2.3	1.4	3.2	31.6	2.6	2.2	3.7	5.2	25.3	0.8
Ref. value	*50.94*	*4.06*	*12.49*	*13.30*	*0.15*	*5.08*	*9.30*	*2.66*	*0.82*	*0.38*	*99.20*
VG-A568 (N=19)											
Average (wt%)	77.56	0.07	12.27	1.11	0.04	0.03	0.44	3.53	4.95	0.01	100.02
RSD%	0.8	69.2	1.9	11.5	142.7	80.5	13.2	4.5	4.7	117.9	0.6
Ref. value	*76.71*	*0.12*	*12.06*	*1.23*	*0.03*	*<0.1*	*0.50*	*3.75*	*4.89*	*<0.01*	*99.62*

Rhyolite glass VG-A568 was used for normalization, basalt glass VG-A99 was used for quality control.
N: number of analyses
RSD: relative standard deviation.
Reference values from *(74)*

Table S3.

Summary of analyses of reference materials VG-A568 (rhyolite glass) and VG-A99 (basalt glass).

Core DP30PC				
Core depth	Rediacarbon age	Calibrated age	Calibrated age	Error
(mm)	(14C age BP)	(cal. yr BP)	(cal. yr)	(95%; ± yr)
789	1290 ± 25	755	1195	135
1792	2535 ± 30	2140	-190	120
2794	3830 ± 40	3635	-1685	195

Table S4.

Gravity core DP30PC ^{14}C dating points based on mixed planktonic foraminifera species *(74)*.

Core GeoB 10709-5

Depth	Mass	Life time (s)	40K	Uncert. 40K	137Cs	Uncert. 137C	210PB	Uncert 210Pb	214PB	Uncert 214Pb	210Pbex	Uncert 210Pbex
cm	g (w.m.)	s	Bq/kg	Bq/kg	Bq/kg	Bq/kg	Bq/kg	Bq/kg	Bq/kg	Bq/kg	Bq/kg	Bq/kg
0.5	41.26	159262	182	6	1.7	0.2	117.4	7.6	11.6	0.7	105.8	7.6
5	20.317	277538	191	7	1.66	0.26	94.5	7	14.3	0.8	80.2	7
8	21.96	269740	270	9	1.64	0.23	86.3	6	14.4	0.8	71.9	6.1
9.5	20.35	174829	271	9	1.88	0.34	74.4	6.6	13.3	0.6	61.1	6.6
10.8	16.76	259245	274	9	1.35	0.33	58.3	5.8	15.5	0.6	42.8	5.8
12	23.62	193746	242	8	0.46	0.11	26.9	3.1	11.2	0.9	15.7	3.2
16	16.885	150623	277	10	<0.49		19.6	4	11.4	0.9	8.2	4.1
20	15.117	230457	257	10	<0.21		20.6	3.7	11.8	1	8.8	3.8
24	13.887	513996	235	8	<0.29		13.4	2.7	10.7	0.8	2.7	2.8
31	24.35	175111	313	10	<0.60		13.6	3.5	13.6	0.5	0	3.5
32	19.363	196518	260	9	0.28	0.2	31.2	3.9	13.3	1	17.9	4
34.8	29.13	254018	282	8	<0.22		15.3	1.9	11.6	0.7	3.7	2

Table S5.

Multicore GeoB 10709-5 ^{210}Pb/^{137}Cs dating. Measured values of activity concentrations. Uncertainties are expressed as 1 standard deviation including counting statistics and detector calibration uncertainty. Values below decision threshold are expressed for 5% type I error probability

Data S1. (separate file)
Elemental composition of all analysed shards of core DP30PC in core section 8 (79 - 179 cm core depth)

Data S2. (separate file)

Multicore GeoB 10709-5, dry bulk density, compaction corrected age and dinoflagellate cyst counts.

Data S3. (separate file)

Gravity core DP30PC counts of dinoflagellate cysts.
